# Food insecurity in Dutch disadvantaged neighbourhoods: a socio-ecological approach

**DOI:** 10.1017/jns.2022.48

**Published:** 2022-06-29

**Authors:** Jolien M. M. Janssen, Laura A. van der Velde, Jessica C. Kiefte-de Jong

**Affiliations:** Department of Public Health and Primary Care/Health Campus The Hague, Leiden University Medical Centre, The Hague, The Netherlands

**Keywords:** Determinants, Explained variance, Food insecurity, Food security, Social ecological model, BMI, body mass index, DHC, Dutch Health Council, FFQ, food frequency questionnaire, FI, food insecure, FS, food secure, IQR, interquartile range, ISCED, International Standard Classification of Education, LUMC, Leiden University Medical Center, MAR, missing at random, MCS, mental component summary, MI, multiple imputation, NNC, Netherlands Nutrition Center, PCS, physical component summary, SEM, social ecological model, SEP, socio-economic position, SF-12, 12-Item Short Form Health Survey, SNAP, Supplement Nutrition and Assistance Program, USDA, United States Department of Agriculture, WMO, ‘Wet medisch-wetenschappelijk onderzoek’, in English: Medical Research Involving Human Subjects Act

## Abstract

Food insecurity is an important public health concern; however, research into this phenomenon within the Netherlands is limited. Food insecurity is not solely related to individual factors, but can also be influenced by various factors in the social and physical environment. Therefore, this study aimed to identify determinants of food insecurity within the personal, social and physical environment, based on the social ecological model (SEM), and to identify their relative importance for experiencing food insecurity. The study population consisted of 307 participants living in disadvantaged neighbourhoods of the Dutch city The Hague, of which approximately one-quarter were food insecure. Participant characteristics showing bivariate associations *P* < 0⋅20 were placed in a predetermined level of the SEM, after which a multivariate logistic regression was performed for each level and the Nagelkerke pseudo *R*^2^ was presented. Determinants of food insecurity were BMI, gross monthly income, highest educational attainment, smoking status, diet quality, employment status, marital status and religion (*P* < 0⋅05). The results showed that 29⋅7 % of the total variance in food insecurity status was explained by all included determinants together. The personal, social and physical environment explained 20⋅6, 14⋅0 and 2⋅4 % of the total variance, respectively. Our findings suggest that determinants within the personal environment are most important for explaining differences in experienced food insecurity. The present study contributes to furthering the knowledge about the relative importance of the personal, social and physical environment, indicating that determinants within the personal environment may be most promising for developing targeted interventions to reduce food insecurity.

## Introduction

Due to various inequalities around the world, 2 billion people are food insecure (FI) worldwide^(1)^. Food insecurity can be defined as an inadequate physical and economic access to adequate foods^(2)^. Contrary to many expectations, food insecurity does not only occur in low- and middle-income countries, but also appears to be a common health issue in high-income countries^(1,2)^. In 2018, as many as 9 % of the citizens of high-income countries were found to experience moderate or severe food insecurity^(2)^. Moreover, previous research including participants from disadvantaged neighbourhoods in the Netherlands found an overall prevalence of food insecurity of 26 %^(3)^. Food security status encompasses both physical and economic access^(4)^. Despite the fact that physical access appears to be easier to achieve the more prosperous the country is, some evidence suggests that there are also countries in Europe, including France and England, where food deserts (i.e., geographic areas with reduced physical access to healthy and affordable food) are still found; in contrast to other European countries such as the Netherlands^(5–8)^.

In addition, it has been found that as the income of the population increases, the emphasis shifts from the quantity to the quality of the diet^(5)^. As a result, diet quality is expected to be more related to the economic differences within higher-income countries (e.g., between cities and even neighbourhoods)^(2,9–11)^. Poorer neighbourhoods are, in addition to often being inhabited by lower-income families, often associated with a high availability of fast food outlets and less healthy foods, which influences the quality and quantity of the available food and may therefore impact the food security status of these families^(12–17)^. Food insecurity is therefore not solely related to the individuals themselves; besides influences from factors in the personal environment, food insecurity can also be influenced by various factors in the social and physical environment. Although previous research has been done into determinants of food insecurity, research into this phenomenon within the Netherlands is still very limited. Previously conducted research by Neter *et al.* found that 70 % of adult Dutch food bank recipients experienced food insecurity^(18)^. Despite the fact that the target group studied by Neter *et al.* is a selection of very underprivileged individuals, it is expected that other underprivileged groups in the Netherlands such as those living in disadvantaged neighbourhoods in urban areas may also be confronted with food insecurity and the associated consequences. Therefore, the aims of the present study were to identify determinants of food insecurity within the personal, social and physical environment among people living in disadvantaged neighbourhoods of the Dutch city The Hague, and to identify the relative importance of these environments for experiencing food insecurity. In the present study, the social ecological model (SEM) was applied to examine associations between determinants within the personal, social and physical environment and food insecurity.

## Methods

### Study design

A cross-sectional analysis was performed. Data collection was done between August 2016 and March 2020. The study was reviewed by the Medical Ethics Committee of Leiden University Medical Centre (LUMC) and confirmed not to be subject to the Medical Research Involving Human Subjects Act (WMO) (P17.164).

### Study population and data collection

Eligible participants were people (1) who lived in or near one of the six predetermined disadvantaged neighbourhoods in The Hague, which were characterised by significant social, physical and economic difficulties that were twice as high as the average in The Hague^(19)^. Furthermore, eligible participants (2) were aged ≥18 years, (3) had at least one child aged <18 years, who lived at home^(3)^ and (4) had sufficient command of the questionnaire language in order to complete the one-time self-administered questionnaire^(3^^)^.

Participants were recruited during two inclusion periods: between August 2016 and June 2018 (inclusion period 1) and between July 2019 and March 2020 (inclusion period 2). Questionnaires were available in Dutch for both inclusion periods. In addition, questionnaires were also available in the English and Turkish language for inclusion period 1. Potential participants were actively approached at public places such as swimming pools, indoor and outdoor playgrounds, churches, community centres and general practices. Participants who experienced difficulties reading and/or writing were offered help filling in the questionnaires by the research team. Written or verbal informed consent was obtained. In both inclusion periods, self-administered questionnaires were used to assess food security status, socio-demographic and lifestyle factors, dietary intake, and to map neighbourhood characteristics. Inclusion period 1 also assessed physical and mental health status.

### Food security status

Food security status was assessed using the 18-item United States Department of Agriculture (USDA) Household Food Security Survey module. The Dutch translated version of the original survey was based on the translation by Neter *et al.*, where the back and forth translation method was applied^(18,20)^. Questions were related to the dietary intake as well as the physical and economic access to (healthy and nutritious) food in the past 12 months of the household in general and the specific experiences of the adults and children in the household^(20,21)^. Affirmative answers were given a score of 1, and non-affirmative answers were given a score of 0. If data were missing, a non-affirmative answer was assumed. Food security scores theoretically ranged between 0 and 18, and were subdivided into the following categories: a score of 0 indicated high food security; a score of 1–2 indicated marginal food security; a score of 3–7 indicated low food security and a score of 8–18 indicated very low food security. In the analyses performed, high food security and marginal food security were classified as food secure (FS) whereas low food security and very low food security were classified as food insecure (FI)^(22)^.

### Social ecological model

**Fig. 1.**
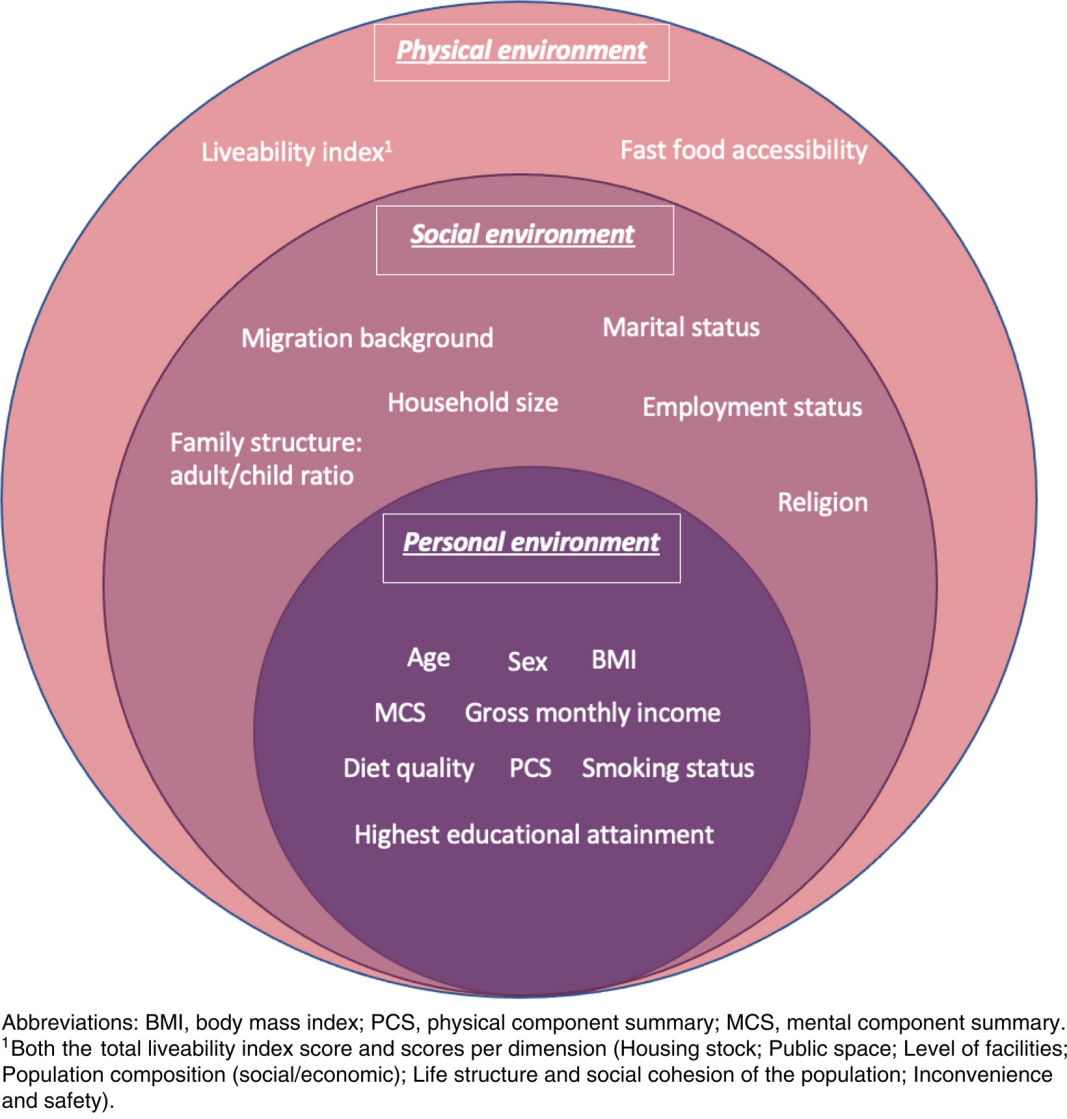
Characteristics of participants that potentially influence the food security status using the social ecological model.

We used the SEM as a theoretical framework to capture influences on food insecurity from different levels within the SEM^(23)^. Therefore, we assigned the participant characteristics described below to the levels of the SEM (Fig. 1)^(23)^. In the present study, participant characteristics were divided into the personal, social and physical levels (based on the intrapersonal, interpersonal, institutional and community levels of the original SEM). The policy level of the SEM was not included in the present study. Within the intrapersonal level of the model, in this study referred to as personal environment, the following biological and personal variables were included: age, sex, BMI, gross monthly income, highest educational attainment, smoking status, physical and mental health and diet quality. Within the interpersonal level, referred to in this study as the social environment, factors related to the individual's social network were included: employment status, marital status, migration background, religion, household size and adult/child ratio. The outer layer used in the present study consisted of the original institutional and community layer of the SEM, which, based on data availability, were merged into an overarching layer; the physical environment. The physical environment consists of variables that are outside the individual and their social network: fast-food accessibility and liveability index. By identifying these different environments separately, the relative importance of each environment on FI could be investigated. A more detailed rationale behind the placement of participant characteristics within the specific environments of the model is presented in Supplementary Table S2.

### Factors included in the SEM

#### Socio-demographic and lifestyle factors

Socio-demographic and lifestyle factors, including: sex, age, height, body weight, household size, marital status, gross monthly income, migration background, religion, highest educational attainment, employment status, and smoking status were obtained using the self-administered questionnaires. To determine current age, participants’ date of birth was subtracted from the date the questionnaire was completed. If participants had not provided their date of birth, their self-reported age in years was used. Body mass index (BMI) was calculated based on the globally used formula: self-reported body weight in kilograms (kg) divided by the self-reported body height in metres square (m^2^)^(24)^. According to the World Health Organization (WHO) cut-off points, BMI was categorised into underweight (BMI <18⋅5 kg/m^2^), normal weight (BMI 18⋅5–24⋅9 kg/m^2^), overweight (BMI 25–29⋅9 kg/m^2^) and obese (BMI ≥ 30 kg/m^2^)^(24)^. To specify the family composition, household size, marital status (two-parent households/single-parent households) and an adult/child ratio (<1, 1, >1) were presented. Household size-adjusted gross monthly income was calculated and dichotomised into above or below the Dutch reference basic needs budget, described in more detail elsewhere^(25,26)^. Migration background was categorised as either having a Western or non-Western migration background^(27)^. Based on data availability, religion was categorised into four categories: Islam, Christianity, not religious and other religion. Highest educational attainment was determined based on the International Standard Classification of Education (ISCED) levels for the education system of the Netherlands, and categorised in either low (≤ISCED 2; less than finished intermediate vocational education), medium (ISCED 3; finished intermediate vocational education) or high (≥ISCED 4; higher than finished intermediate vocational education)^(28)^. If the highest educational attainment of the participant was not in the Netherlands, the country-specific ISCED levels were used to determine the corresponding educational level^(28)^. We further assessed current employment (yes/no) and current smoking status (yes/no).

#### Dietary intake

A short food frequency questionnaire (FFQ) was used to determine dietary intake and subsequently calculate a total diet quality score ranging from 0 to 100, based on adherence to current national dietary intake recommendations from the Dutch Health Council (DHC) and the Netherlands Nutrition Center (NNC)^(29–31)^. Higher scores indicate better adherence to the recommendations. Detailed information of the dietary intake assessment and diet quality score calculation is provided in Supplementary Methods 1 and Table S1.

#### Fast-food food environment

In order to determine the fast-food accessibility and availability, the shortest distance from the participants’ home address (using their six-digit postal code (or four-digit if the six-digit postal code was not available)) to the nearest fast-food outlet and the number of fast-food outlets within a radius of both 500 and 1000 m from the participants’ home address were reported. Data related to fast-food outlets were extracted from the Locatus database^(32)^. A more detailed description of the methods used is described elsewhere^(33)^.

#### Liveability index

Liveability of the neighbourhood was assessed using the liveability index (in Dutch: ‘leefbaarometer’), drawn up by the Ministry of the Interior and Kingdom Relations^(34,35)^. This index ranges from very poor (1) to outstanding (9), based on various factors representing six dimensions: Housing stock; Public space; Level of facilities; Population composition (social/economic); Life structure and social cohesion of the population; Inconvenience and safety. The liveability index was linked using the 4-digit postal code of the participants. In addition to a total liveability index score, the scores (deviation score from the national average) per dimension were also reported in order to gain more insight into the influence of the separate dimensions.

#### Physical and mental health status

The 12-Item Short Form Health Survey (SF-12) was used in order to assess the participants’ mental and physical health^(36)^. A more detailed description of the scoring procedure of the SF-12 is provided elsewhere^(37)^. The SF-12 score obtained was divided into two sub-scores: the mental component summary (MCS) and physical component summary (PCS). Both scores theoretically ranged between 0 and 100, with higher mental and physical component scores corresponding to better mental and physical health.

### Statistical analyses

Descriptive statistics of all participant characteristics were presented according to the levels of the SEM for the total population and separately for FS and FI participants, using the median and interquartile ranges (IQR) for continuous variables, and frequencies and percentages for categorical variables. Furthermore, bivariate associations between food security status (food secure *v*. food insecure) and participant characteristics were assessed using the *χ*^2^ test or *t*-test, as appropriate. Participant characteristics were placed in the predetermined levels of the SEM when bivariate associations showed *P* < 0⋅20 (Fig. 1)^(38)^. For each level of the SEM, a multivariate logistic regression was performed including all remaining variables within the level and food insecurity score as outcome. The Nagelkerke pseudo *R*^2^ was presented to compare the explained variance in food insecurity for each level. Furthermore, multiple imputation (MI) was applied, using the fully conditional specification method (Markov chain Monte Carlo method), in order to manage missing data. Based on the percentage of missing data, *n* 38 imputed datasets were generated^(39)^. Before the MI procedure was conducted, pattern analyses were performed and missing data was assumed to be missing at random (MAR). Logistic regression models were used for categorical variables, and predictive mean matching was chosen as model for variables that were not normally distributed. An overview of all imputed variables, as well as the auxiliary variables, is shown in Supplementary Table S3. Since the imputed data provided similar results to the unimputed data, pooled results of the imputed data were reported (Supplementary Tables S4 and S5). Data analyses were performed using IBM SPSS Statistics, version 25 (IBM Corp., 2012, Armonk, NY). For all statistical analyses, a two-sided *P*-value of 0⋅05 and bivariate association of *P* < 0⋅20 demonstrated a statistically significant association.

## Results

Complete data were collected from 307 participants, of whom 229 were from inclusion period 1. In total, 233 (75⋅9 %) participants were FS and 74 (24⋅1 %) participants were FI (Table 1).

**Table 1.** Participant characteristics stratified by food security status (*N* 307)

Characteristics	Median (IQR) or *N* (%)			*P*-value
	Total study participants (*N* 307)	Food secure participants (*N* 233)	Food insecure participants (*N* 74)	
**Personal environment**				
Age, years	37⋅4 (33⋅4; 42⋅1)	36⋅9 (32⋅9; 41⋅3)	39⋅0 (34⋅0; 43⋅7)	0⋅105*
Sex, %				0⋅491
Male^a^	39 (12⋅7)	28 (12⋅0)	11 (14⋅9)	
Female	268 (87⋅3)	205 (88⋅0)	63 (85⋅1)	
Self-reported BMI^b^, kg/m^2^	27⋅2 (24⋅1; 30⋅5)	26⋅9 (24⋅0; 29⋅8)	28⋅6 (25⋅5; 32⋅6)	0⋅016**
Weight status^c^, %				0⋅021**
Normal weight; BMI 18⋅5–24⋅9^a^	96 (31⋅3)	79 (33⋅9)	17 (23⋅0)	
Overweight; BMI 25–29⋅9	124 (40⋅4)	97 (41⋅6)	27 (36⋅5)	
Obese; BMI ≥ 30	87 (28⋅3)	57 (24⋅5)	30 (40⋅5)	
Gross monthly income, %				<0⋅001****
Below basic needs budget^a^	182 (59⋅3)	122 (52⋅4)	60 (81⋅1)	
Above basic needs budget	125 (40⋅7)	111 (47⋅6)	14 (18⋅9)	
Highest educational attainment, %				0⋅002***
Low; ≤ISCED 2^a^	110 (35⋅8)	73 (31⋅3)	37 (50⋅0)	
Medium; ISCED 3	111 (36⋅2)	84 (36⋅1)	27 (36⋅5)	
High; ≥ISCED 4	86 (28⋅0)	76 (32⋅6)	10 (13⋅5)	
Current smoking, %				0⋅005***
Yes^a^	51 (16⋅6)	31 (13⋅3)	20 (27⋅0)	
No	256 (83⋅4)	202 (86⋅7)	54 (73⋅0)	
Physical and mental health status				
PCS; range 0–100	44⋅6 (36⋅1; 53⋅5)	44⋅5 (36⋅9; 53⋅6)	42⋅5 (33⋅8; 49⋅5)	0⋅099*
MCS; range 0–100	46⋅3 (36⋅0; 54⋅5)	46⋅2 (36⋅6; 55⋅3)	43⋅2 (33⋅8; 50⋅4)	0⋅068*
Diet quality; range: 0–100	56⋅3 (50⋅1; 67⋅0)^d^	56⋅5 (51⋅0; 68⋅1)	55⋅7 (44⋅5; 63⋅8)	0⋅022***
Population-specific median diet quality score, %				0⋅442
Low diet quality; ≤56⋅3^a^	154 (50⋅2)	114 (48⋅9)	40 (54⋅1)	
High diet quality; >56⋅3	153 (49⋅8)	119 (51⋅1)	34 (45⋅9)	
**Social environment**				
Currently employed, %				0⋅004***
Yes^a^	141 (45⋅9)	118 (50⋅6)	23 (31⋅1)	
No	166 (54⋅1)	115 (49⋅4)	51 (68⋅9)	
Marital status, %				0⋅034**
Two-parent household^a^	218 (71⋅0)	173 (74⋅2)	45 (60⋅8)	
Single-parent household	89 (29⋅0)	60 (25⋅8)	29 (39⋅2)	
Migration background, %				0⋅067*
Western^a^	63 (20⋅5)	53 (22⋅7)	10 (13⋅5)	
Non-Western	244 (79⋅5)	180 (77⋅3)	64 (86⋅5)	
Religion, %				0⋅021**
Islam^a^	184 (60⋅0)	143 (61⋅4)	41 (55⋅4)	
Christianity	51 (16⋅7)	30 (12⋅9)	21 (28⋅4)	
Not religious	45 (14⋅7)	38 (16⋅3)	7 (9⋅4)	
Other religion	27 (8⋅8)	22 (9⋅4)	5 (6⋅8)	
Household size, *N*	4⋅0 (3⋅0; 5⋅0)	4⋅0 (3⋅0; 5⋅0)	4⋅0 (3⋅0; 5⋅0)	0⋅360
Adult/child ratio	1⋅0 (0⋅5; 1⋅0)	1⋅0 (0⋅5; 1⋅0)	0⋅7 (0⋅5; 1⋅0)	0⋅380
Adult/child ratio, %				0⋅796
<1^a^	153 (49⋅8)	114 (48⋅9)	39 (52⋅7)	
1	106 (34⋅5)	81 (34⋅8)	25 (33⋅8)	
>1	48 (15⋅6)	38 (16⋅3)	10 (13⋅5)	
**Physical environment**				
Fast-food accessibility^e^				
Shortest distance to the nearest fast-food outlet, m	136⋅3 (109⋅0; 215⋅8)	135⋅4 (109⋅0; 215⋅2)	138⋅8 (108⋅5; 221⋅3)	0⋅973
Number of fast-food outlets within a radius of 500 m	12⋅0 (6⋅0; 18⋅0)	12⋅0 (6⋅3; 18⋅0)	12⋅0 (6⋅0; 17⋅0)	0⋅727
Number of fast-food outlets within a radius of 1000 m	48⋅5 (24⋅0; 62⋅0)	49⋅0 (24⋅0; 60⋅8)	47⋅0 (25⋅0; 65⋅0)	0⋅923
Liveability index score^e^; range: 1–9^g^	3⋅0 (3⋅0; 4⋅0)	3⋅0 (3⋅0; 4⋅0)	3⋅0 (3⋅0; 4⋅0)	0⋅678
Liveability index^f^^,^^g^^,^^h^				
Dimension: Housing stock	0⋅03	0⋅03 (−0⋅03; 0⋅61)	0⋅02 (−0⋅11; 0⋅05)	0⋅055*
Dimension: Population composition	−0⋅03	−0⋅34 (−0⋅39; −0⋅24)	−0⋅29 (−0⋅36; −0⋅23)	0⋅336
Dimension: Level of facilities	0⋅24	0⋅24 (0⋅15; 0⋅29)	0⋅24 (0⋅14; 0⋅29)	0⋅865
Dimension: Inconvenience and safety	−0⋅28	−0⋅29 (−0⋅32; −0⋅17)	−0⋅28 (−0⋅32; −0⋅20)	0⋅665
Dimension: Public space	−0⋅16	−0⋅16 (−0⋅22; −0⋅08)	−0⋅16 (0⋅22; −0⋅08)	0⋅516

Abbreviations: IQR, interquartile range; N, number; BMI, body mass index; ISCED, International Standard Classification of Education; PCS, physical component summary; MCS, mental component summary; m, metres.

a Reference category.

b Calculated based on the self-reported body weight in kilograms (kg) divided by the self-reported body height in metres square (m^2^).

c Since only two participants were classified as underweight, this category was merged with normal weight, and therefore only normal weight, overweight and obesity were reported.

d Population-specific median diet quality score.

e Data available for *N* 214.

f Data available for *N* 279.

g Index range: (1) very poor, (2) poor, (3) very unsatisfactory, (4) unsatisfactory, (5) satisfactory, (6) more than satisfactory, (7) good, (8) very good and (9) outstanding.

h Reported as deviation scores from the national average (national average = 0).

*P < 0⋅20, **P < 0⋅05, ***P < 0⋅01, ****P < 0⋅001 for the difference between food-insecure and food-secure households.

### Participant characteristics in the personal, social and physical environment

The median (IQR) age of the participants was 37⋅4 (33⋅4; 42⋅1) years, the majority of participants were female (87⋅3 %), and more than two-third of the participants were either overweight or obese (Table 1). FI participants had a higher median BMI (28⋅6 (25⋅5; 32⋅6) *v*. 26⋅9 (24⋅0; 29⋅8)), and showed a higher prevalence of obesity (40⋅5 *v*. 24⋅5 %), compared to FS participants. Of the participants, 59⋅3 % had a gross monthly income below the Dutch basic needs budget, 35⋅8 % was low educated and 16⋅6 % were current smokers. FI participants more often had a monthly gross income below the Dutch basic needs budget (81⋅1 *v*. 52⋅4 %) compared to FS participants. Median PCS and MCS scores were 46⋅3 (36⋅0; 54⋅5) and 44⋅6 (36⋅1; 53⋅5) out of 100, respectively, where higher scores indicate a better health. Both the MCS and PCS scores were found to be lower for FI participants compared to FS participants. Furthermore, the median total diet quality score was 56⋅3 (50⋅1; 67⋅0) out of 100 (Table 1). The majority of participants were currently not employed (54⋅1 %), lived in a two-parent household (71⋅0 %), had a non-Western migration background (79⋅5 %) and were Islamic (60⋅0 %). FI participants were more often current smokers (27⋅0 *v*. 13⋅3 %), currently unemployed (68⋅9 *v*. 49⋅4 %) and living in a single-parent household (39⋅2 *v*. 25⋅8 %) compared to FS participants. In addition, the median total liveability score of the neighbourhood was found to be very unsatisfactory, and median shortest distance to the nearest fast-food outlet was 136⋅3 (109⋅0; 215⋅8) m. Within the physical environment, only the housing stock dimension of the liveability index showed a bivariate association of *P* < 0⋅20 and was 0⋅03 higher than the median score in the Netherlands, with scores found to be lower for FI participants than for FS participants.

### Relative importance of the personal, social and physical environment for explaining food insecurity

Table 2 shows the results from a logistic regression analysis indicating the odds of a participant being FI for each determinant variable (with bivariate association *P* < 0⋅20) within the pre-specified environments. Determinants within the personal environment indicate that individuals with a higher BMI, who had an income below the basic needs budget, were low educated (≤ISCED 2), were current smokers, had lower PCS and MCS scores, and lowest diet quality scores were at increased risk of food insecurity. Determinants within the social environment indicate that individuals who were currently not employed, lived in a single-parent household, had a non-western migration background and were Christians (compared to Islam (=reference group)), were more likely to be FI. Individuals with a relatively low score for the housing stock dimension were at increased risk of food insecurity.

**Table 2.** Logistic regression analysis of the associations between food insecurity status and participant characteristics within the specific layers of the social ecological model (*N* = 307)

	Adjusted OR^a^	95 % CI	*P*-value	Nagelkerke pseudo *R*^2^
**Personal environment**				0.206
Self-reported BMI^b^, kg/m^2^	1.07	(1.00; 1.13)	0.04*	
Gross monthly income, %				
Below basic needs budget	Reference			
Above basic needs budget	0.34	(0.17; 0.69)	<0.01**	
Highest educational attainment, %				
Low; ≤ ISCED 2	Reference			
Medium; ISCED 3	0.88	(0.46; 1.69)	0.70	
High; ≥ ISCED 4	0.48	(0.21; 1.15)	0.10	
Current smoking, %				
Yes	Reference	(0.21; 0.91)	0.03*	
No	0.44			
Physical and mental health				
PCS; range 0–100	0.99	(0.85; 1.03)	0.54	
MCS^c^; range 0–100				
≤35	Reference			
36–46	1.14	(0.39; 3.35)	0.82	
47–54	0.98	(0.34; 2.78)	0.97	
≥55	0.57	(0.19; 1.69)	0.31	
Diet quality^c^; range: 0–100				
≤49	Reference			
50–55	0.53	(0.28; 1.44)	0.28	
56–66	0.89	(0.40; 1.97)	0.77	
≥67	0.53	(0.23; 1.23)	0.14	
**Social environment**				0.140
Currently employed, %				
Yes	Reference			
No	2.23	(1.21; 4.08)	0.01*	
Marital status, %				
Two-parent household	Reference			
Single-parent household	1.69	(1.10; 3.13)	0.10	
Migration background, %				
Western	Reference			
Non-Western	2.53	(1.02; 5.90)	0.03*	
Religion, %				
Islam	Reference			
Christianity	3.12	(1.61; 6.18)	<0.01**	
Not religious	0.86	(0.34; 2.19)	0.73	
Other religion	0.37	(0.13; 1.06)	0.06	
**Physical environment**				0.024
Liveability index^d^^,^^e^				
Housing stock	0.33	(0.25; 0.43)	<0.001***	
Overall model				0.297

Abbreviations: OR, odds ratio; CI, confidence interval; BMI, body mass index; ISCED, International Standard Classification of Education; PCS, physical component summary; MCS, mental component summary.

a Indicating the odds of a participant being FI for each determinant variable.

b Calculated based on the self-reported body weight in kilograms (kg) divided by the self-reported body height in metres square (m^2^).

c Categories based on quartiles.

d Data available for *N* 279.

e Reported as deviation scores from the national average (national average = 0).

*P < 0.05, **P < 0.01, ***P < 0.001 for the difference between food-insecure and food-secure households.

Table 2 also shows the Nagelkerke pseudo *R*^2^ for the full model and for the personal and social environment separately. The overall model showed a Nagelkerke pseudo *R*^2^ of 0⋅297 indicating that 29⋅7 % of the total variance in food insecurity status was explained by the included determinants. The personal, social and physical environment explained 20⋅6, 14⋅0 and 2⋅4 % of the total variance, respectively.

## Discussion

About a quarter of the study population experienced food insecurity. Food security status was significantly associated with BMI, gross monthly income, highest educational attainment, smoking status, diet quality, current employment status, marital status and religion. Based on the levels of the SEM, our results indicate that determinants within the personal environment explained most of the variance in food insecurity (20⋅6 %), determinants within the social environment explained 14⋅0 % of the variance in food insecurity, and determinants within the physical environment barely contributed to explaining food insecurity.

The prevalence of approximately one-quarter of participants experiencing food insecurity found was in accordance with previously conducted studies in Europe, i.e., 25⋅0 % in low socio-economic position (SEP) groups in the United Kingdom^(40,41)^. In addition, the prevalence of food insecurity found in the present study was comparable to the average European food insecurity prevalence of 25⋅7 % found in a study that included 38⋅194 citizens of 39 European countries^(42)^. In accordance with our hypothesis and earlier conducted research, BMI, gross monthly income, highest educational attainment, smoking status, employment status, marital status, diet quality and religion were found to be significant determinants of food insecurity.

Since a healthy diet is generally more expensive than an unhealthy diet and the prices of healthy products rising more than the prices of unhealthy products, people increasingly have to spend a larger part of their budget on healthy foods, which can ultimately lead to less healthy dietary choices and food insecurity among lower SEP populations^(43–47)^. Unhealthy dietary choices have been associated with inflammation and weight gain^(48,49)^. Since a healthy diet is generally more expensive than an unhealthy diet, a vicious circle may arise in which i.a. BMI, dietary intake and food insecurity continue to reinforce each other, whereby dietary factors mediate these relationships among lower SEP populations^(43–45,50)^. In addition, a high BMI is associated with a reduced job opportunity and therefore people with a high BMI may be less likely to have steady income which may lead to a lower income^(51)^. Furthermore, as expected as food insecurity and low income are closely related, our results showed that people with a more difficult financial situation have a higher risk of experiencing food insecurity^(52)^. Similarly, smoking, unemployment and living in a single-parent household, all associated with low SEP, appear to increase the risk of food insecurity, for example due to less money being available for food and experiencing more stress^(53–55)^. Although previous studies have shown that visiting religious meeting places improves the social network, results of the present study showed that participants who were not religious or had another religion than Christianity or Islam were less likely to be FI than Muslims or Christians^(56–58)^. This may be because Muslims and Christians are expected to share their food with others based on their faith, even if they are still hungry themselves^(59)^. In addition, in the present study, Christians were found to experience food insecurity more often than Muslims. This could possibly be explained by the differences in social support systems within different religions^(60)^. It should further be noted that the different religions have not been examined in comparable proportions in the present study, which may have influenced the results. Moreover, as a relatively small proportion of the participants were Christian, the sampling method may also have played a role. One of the public places where potential participants were actively approached was a church that also functioned as a food bank, targeting people experiencing financial difficulties and therefore at higher risk of food insecurity. This may have led to an overestimation of the number of Christians experiencing food insecurity.

Although previous research suggests that the SEM is a very suitable theoretical framework to capture influences of different levels on health-related behaviour^(23)^, our result was that only 29⋅7 % of the total variance in food insecurity was explained by the determinants included in the model. However, these findings should be interpreted with caution, given the possible lack of included variables in the present study that may also be important for explaining food insecurity, such as, for example, health literacy and financial management skills^(61–64)^. The relatively low explained variance may also be due to the interrelationship of determinants. Within current research, determinants within the personal and social environment (and therefore two out of the three investigated environments of the SEM) were found to be significantly associated with food insecurity. Based on the explained variance reported in the present study, the personal environment appeared to be the most important environment for explaining food insecurity. This is in accordance with previous research, which showed that factors from the social environment influence the reinforcement or moderation of the associations found between personal determinants and food insecurity^(65,66)^. This finding may also be explained by the fact that more data were available and therefore more determinants were included in this environment. So far, interventions to combat food insecurity have mainly focused on determinants from the personal environment, as these interventions often seem to focus on providing information or distributing food (vouchers), in order to improve the diet-related knowledge and diet quality of individuals^(67)^. Although our findings indicate that determinants within the personal environment are indeed most important for explaining food insecurity, and may therefore be a promising target for interventions, previous literature shows that policy-level interventions (e.g., the US Supplement Nutrition and Assistance Program (SNAP)) appear to have a greater impact on reducing food insecurity than personal or community-level interventions, (e.g., food banks). Psychosocial factors such as shame may play a role in this^(68)^. It should however be noted that is very difficult to intervene at the policy level and therefore this layer of the SEM was beyond the scope of the present study, as we aimed to contribute to identifying high risk groups for food insecurity and subsequently contribute to preventive mapping of these high risk groups and provide targets for interventions to improve their food security. None of the determinants within the physical environment showed a significant bivariate association. This may be explained by the fact that both FS and FI participants lived in the disadvantaged neighbourhoods of The Hague and therefore may have presented a relatively homogeneous group in terms of their physical environment. Larger differences can be expected when looking at differences in neighbourhood characteristics at the national level. Future studies may, by including a larger study population and study area, further examine determinants of food insecurity in the physical environment.

The present study is strengthened by the inclusion of an extensive set of socio-demographic and lifestyle factors allowing for many determinants to be included in each environment of the SEM.

However, it should be noted that other determinants that were not included in the current analyses may be important for explaining food insecurity in the different layers, as the total explained variance was limited.

Despite the use of two inclusion periods, the study populations and sampling areas and techniques were similar during the two inclusion periods. Moreover, during both inclusion periods, participants were included during different seasons of the year. As a result, minimal bias is expected due to the influence of inclusion periods and seasons on perceived food insecurity.

Furthermore, we performed MI to reduce potential bias associated with missing data^(39)^.

However, the study should be interpreted in light of its limitations as well. First, the 18-item USDA Household Food Security Survey Module, which is regarded as the golden standard for assessing food insecurity in the Western countries, has not yet been validated for the Dutch language and context^(53)^. As a result, misclassification by food security status cannot be ruled out. Furthermore, since missing data for food security status were reported as non-affirmative answers, it is possible that food security status was underestimated in the current study population. The same holds for dietary quality. However, as missing data for these types of questions often indicates that the situation does not apply (food insecurity) or the food is not consumed (diet quality), this method was preferred over MI techniques and is expected to provide a better estimate of food security and diet quality ranking among the participants than would be obtained using MI^(69)^. Finally, our results may not be generalisable to the total Dutch population, as we conducted the study in disadvantaged neighbourhoods of the Dutch city The Hague. This may have resulted in a higher prevalence of food insecurity compared to the total Dutch population, however it is not expected to have influenced the found associations presented in this study^(70)^.

## Conclusion

In conclusion, various determinants were shown to be associated with experiencing food insecurity. Current results indicate that determinants within the personal environment appear to be the most important for explaining differences in experienced food insecurity. Determinants within this environment should therefore be central to the preventive mapping of high risk groups for food insecurity and subsequently intervene to improve their food security. Future studies should confirm current results in different populations, as well as include additional determinants such as psychosocial determinants to investigate other potential determinants that may be important for explaining differences in food insecurity status. Hereby, a higher proportion of the variance in food security can possibly be explained and thus the relative importance of the different environments can be examined more accurately. All in all, the present study contributes to furthering the knowledge about the relative importance of the different environments within the SEM, thereby indicating potential targets for developing targeted interventions to reduce food insecurity.

## Supplementary material

For supplementary material accompanying this paper visit https://doi.org/10.1017/jns.2022.48. click here to view supplementary material
