# Supplementary material for: Food insecurity in Dutch disadvantaged neighbourhoods: a socio-ecological approach
Source: J Nutr Sci. 2022 Jun 29;11:e52. doi: 10.1017/jns.2022.48 (PMC9257769; doi:10.1017/jns.2022.48)
Supplement: Supplementary file 1 [file S2048679022000489sup001.docx]

**Supplementary materials**

**Supplementary methods 1** Dietary intake

In both inclusion periods we used a short FFQ to determine dietary intake and subsequently calculate a total diet quality score, based on adherence to current national dietary intake recommendations from the Dutch Health Council (DHC) and the Netherlands Nutrition Center (NNC) (1-3). A total diet quality score, reflecting adherence to current Dutch dietary guidelines, was calculated in both study phases based on 6 and 13 dietary components respectively *(*diet quality score study phase 1: (4)***;*** diet quality score study phase 2: ***Supplementary table 1)***. To construct the total diet quality score, each component was assigned a score ranging from 0 to 10, where a score of 0 represented minimal adherence to the Dutch dietary guidelines and a score of 10 represented maximum adherence to the Dutch dietary guidelines (study phase 1: (4)***;*** study phase 2: ***Supplementary table 1).*** If data were missing for a dietary component, the least favorable outcome was assumed. Scores of all components were summed, resulting in a total theoretical diet quality score ranging between 0 and 60 for the first study phase and ranging between 0 and 130 for the second study phase. Both scores were converted into a score ranging from 0-100 to obtain a comparable range between the two study phases. A higher total diet quality score corresponded to a higher adherence to the Dutch dietary guidelines for a healthy diet. An overview of the dietary components included with associated national dietary recommendations per study phase, contribution percentages and the component specific scoring system are presented in the ***Supplementary table 1*** and described in the study van der Velde et al. (2020) (4)***.***

|  | Supplementary table 1 Dietary components with associated current national dietary recommendations, contribution percentages and scoring per component for study phase 2 | | | | | | |
| --- | --- | --- | --- | --- | --- | --- | --- |
|  | **Dietary**  **component** | **Dietary recommendations by the Dutch Health Council and/or the Netherlands Nutrition Centre** | **% contribution to the dietary component score** | **Units** | **Dietary component score**  ***MINIMUM SCORE MAXIMUM SCORE***  ***(=0 POINTS)*  5 *POINTS (=10 POINTS)*** | | |
|  | **Vegetables** | Consume at least 200 grams of vegetables daily | 100 | g/d | 0 | *Continuous* | ≥200 |
|  | **Fruit** | Consume at least 200 grams of fruit daily | 100 | p/d | 0 | *Continuous* | ≥ 2 |
|  | **Legumes** | Consume one serving legumes a week | 100 | g/w | 0 | *Continuous* | ≥ 135^1^ |
|  | **Unsalted nuts** | Consume at least 15 grams of unsalted nuts daily | 100 | g/d | 0 | *Continuous* | ≥ 15 |
|  | **Fish** | Consume one serving of fish weekly, preferably fatty fish | 50  50 | s/w  - | 0  No fish consumed | <1  Lean or both lean and fatty fish | ≥ 1  Mostly fatty fish |
|  | **Grain products** | Consume at least 90 grams of whole grain products daily  Replace refined grain products by whole grain products | 50  50 | g/d  - | 0  Mostly refined | *Continuous*  Both refined and whole grain | ≥ 90  Mostly whole grain |
|  | **Dairy** | Consume 2-3 servings of dairy daily | 50  50 | s/d  - | 0  Full fat dairy products | *Continuous*  Both whole dairy products and (semi)-skimmed dairy products | ≥ 2  (Semi)-skimmed dairy products |
|  | **Tea** | Consume 3-4 cups of green/black tea a day | 100 | s/d  - | <1 **AND** mostly green/black tea  <3 **AND** both herbal tea and green/black tea  Not consumed  Mostly herbal tea | 1 to 2 **AND** mostly green/black tea  ≥ 3 **AND** both herbal tea and green/black tea | ≥ 3 **AND** mostly green/black tea |
|  | **Coffee** | Replace unfiltered coffee by filtered coffee | 100 | - | Boiled coffee, cafetière coffee, Greek coffee, Turkish coffee | Vending-machine coffee^2^, coffee from cups and espresso | Not consumed or filter coffee, coffee from pads and instant coffee |
|  | **Oils and fats** | Replace butter, hard margarines and cooking fats by soft margarines, liquid cooking fats, and vegetable oils | 50  50 | - | Butter, hard margarines  Butter on bread or bread is not buttered at all | Both butter, hard margarines and oils and soft margarines  Semi-skimmed butter or hard margarine on bread | Oils and soft margarines  Diet margarine on bread |
|  | **SCBs** | Minimize consumption of sugar-containing beverages. | 100 | s/d | ≥ 1 | <1 | 0 |
|  | **Savory snacks** | For products outside the Wheel of Five: consume an item from the daily selection no more than three to five times per day, and something from the weekly selection no more than three times a week | 50  50 | lrg s/w  sml s/d | ≥ 3  > 3 | <1 to 2  *Continuous* | 0  0 |
|  | **Sweet snacks** | For products outside the Wheel of Five: consume an item from the daily selection no more than three to five times per day, and something from the weekly selection no more than three times a week | 100 | s/w | ≥ 3 | <1 to 2 | 0 |

*Abbreviations: SCB, sugar-containing beverages; g/d, grams per day; p/d, pieces per day; s/w, servings per week; s/d, servings per day; lrg s/w, large servings per week; sml s/d, small servings per day.*

*^1^The Netherlands Nutrition Centre indicates that one serving of legumes corresponds to 135 grams of legumes (1).*

*^2^Vending-machine coffee can be either filtered coffee or unfiltered coffee. Since the filter used by the vending- machine is not known, the cafestol level is assumed to be moderate (1).*

Supplementary table 2 Rationale for the placement of participant characteristics within the specific layers of the SEM

|  | ***Rationale*** |
| --- | --- |
| *Age* | Knowledge about a healthy diet varies by age. Middle-aged adults (45+) appear to have the highest dietary knowledge and can therefore make better decisions with regard to diet quality (5, 6). In addition, older adults more often have a permanent contract compared to younger adults and therefore have greater income security. This leads to a lower risk of food insecurity (7). |
| *Sex* | In general, females have a more important role in taking care of their family and in preparing and purchasing food than males (8, 9). In addition, males have less dietary knowledge than females. This allows females to make better choices regarding a healthy diet and possibly less likely to be FI than males (5, 10, 11). |
| *BMI* | A high BMI is associated, among other things, with a low income (12). In addition, it is associated with a reduced job opportunity and therefore are people with a high BMI less likely to have steady income. Job discrimination mainly occurs among obese females (13). As a result, people with a higher BMI are more likely to be FI. |
| *Employment status* | Unemployment has negative consequences on your mental health, and causes a lot of stress and a higher chance of depressive symptoms. As a result, individuals may take less care of themselves, decrease social contact and have therefore a higher risk of food insecurity (14-16). In addition, individuals in current employment also receive social support from their colleagues, which allows them to receive additional assistance if needed, thereby reducing their risk of food insecurity (17). |
| *Gross monthly income* | A higher income can reduce the risk of food insecurity. For example, individuals with a high income need to set fewer priorities with regard to their budget, so that they will have more money to spend on sufficient and healthy food and will less likely experience stress related to food security. In addition, a low income more often leads to a reduced quantity and quality of food and therefore more food insecurity (18). |
| *Highest educational attainment* | Individuals with a higher education generally also have better dietary knowledge, so that they know better what good quality food is, but also have better financial knowledge, exhibit more saving behavior and can therefore make better choices in terms of nutrition within their expenditure (19, 20). Moreover, the highly educated more often have a paid job and the salary of the highly educated is generally higher than for the lower educated, so that they have more money available to avoid food insecurity (5, 19, 21). |
| *Smoking status* | Smoking is often associated with having a low SEP. People with a low SEP have less money to spend on food and therefore a greater risk of food insecurity (22, 23). Furthermore, smoking is an extra expense, resulting in less budget for food and smokers appear to have higher lifetime health care costs (24). |
| *Migration background* | Having family dinners has been associated with healthier dietary behavior (25). Besides, in certain cultures and countries, people often eat together with their families. By sharing food and enhancing social networks, the risk of food insecurity can be reduced. Moreover, individuals of non-native descent may be less able to apply for help (e.g. food banks), due to barriers such as language problems, and therefore experience a higher risk of food insecurity (26). In addition, Dutch natives and others with a western migration background have on average a higher income and therefore a lower risk of food insecurity (27). |
| *Religion* | Individuals build an extra social network at a church or mosque. In addition to this extra social support, places such as a church or mosque often offer free meals, reducing people's risk of food insecurity (28-30). |
| *Physical and mental health (SF-12)* | Mental problems can lead to a lower dietary intake, but also a reduced quality of diet due to increased stress and a lower energy level. Physical limitations can make it more difficult to run errands, etcetera. (31). |
| *Diet quality* | A healthy diet is more expensive than an unhealthy diet. Furthermore, between 2007 and 2017, prices of healthy foods have risen higher than the prices of unhealthy foods (32). As a result, people increasingly have to spend a larger part of their budget on a diet, which can ultimately lead to food insecurity. |
| *Marital status* | Married or cohabiting couples may have more employed adult members in the household and are therefore less likely to be FI than single parents (33). Moreover, a partner probably also provides a larger social network and provides mental support, for example (34). Females living in single-parent households experience more often multiple economic and time-related barriers to eating healthy compared to males living in single-parent households (35). This may be because males more often have a full-time job than females, and are therefore more able to support the family financially (36). |
| *Household size* | The larger the household, the more people have to be supported by a certain income. This increases the risk of food insecurity (37, 38). |
| *Family structure:*  *adult/child ratio* | Parents sacrifice their own dietary quality to ensure adequate dietary quality for their children. The lower the ratio, the more we expect parents to potentially sacrifice their own dietary quality (39). |
| *Fast-food accessibility* | Poorer neighborhoods are often associated with the high availability of fast food and less healthy foods (40, 41). In addition, poorer neighborhoods are often associated with lower income families, which is often associated with a reduced quantity and quality of food and thus a greater risk of food insecurity (18). |
| *Liveability index* | Poorer neighborhoods are often associated with the high availability of fast food and less healthy food (40, 41). In addition, these poorer neighborhoods are often seen as less safe and associated with more litter and less greenery, so that people go outside less often and are therefore less physically active and experience a poorer (mental) health (42-44). |

Supplementary table 3 Overview of the multiple imputation procedures

| ***Total multiple imputation procedure*** | |
| --- | --- |
| *Inclusion periods* | Inclusion period 1 and 2 |
| *Software used for MI* | IBM SPSS Statistics, version 25 |
| *Number of imputations* | 38 |
| *Imputation method and key settings* | Fully conditional specification;  Maximum iterations: 10 |
| *Variables included in the MI procedure in order to be imputed and used as a predictor to impute missing data.* | Leeftijd_totaal Geslacht Lengte Gewicht Total_Bruto_inkomen_S3 Burgerlijke_staat_samen  Opleidingsniveau_samen Migratieachtergrond_Westers_nietWesters Huidig_betaalde_baan  Verleden_betaalde_baan Total_Huidig_Roken Total_Verleden_Roken Religie_4cat Gem_beweging_minuten_dag SF_1 SF_2a SF_2b SF_3a SF_3b SF_4a SF_4b SF_5 SF_6a SF_6b SF_6c SF_7 |
| *Variables additionally included in the imputation method as a predictor to impute missing data (to increase the validity of MAR assumption).* | FoodSecurity_1 FoodSecurity_2 FoodSecurity_3 FoodSecurity_4 FoodSecurity_4a FoodSecurity_5 FoodSecurity_6 FoodSecurity_7 FoodSecurity_8 FoodSecurity_8a FoodSecurity_9 FoodSecurity_10 FoodSecurity_11 FoodSecurity_12 FoodSecurity_13 FoodSecurity_14 FoodSecurity_14a FoodSecurity_15  FoodSecurity_16 |
| *Model type used for not normally distributed variables* | Predictive mean matching |
| *Model type used for categorical variables* | Logistic regression |

*Abbreviations: MI, multiple imputation; MAR, missing at random.*

| Supplementary table 4 Socio-demographic and lifestyle factors stratified by food security status in unimputed and imputed data (N=307) | | | | | | | | |  |
| --- | --- | --- | --- | --- | --- | --- | --- | --- | --- |
|  | **Missing in unimputed data** | **Unimputed data** | | | **Imputed data** | | | | |
|  | **N (%)** | **Median (IQR) or N (%)** | | | **Median (IQR) or N (%)** | | | | |
| Characteristics |  | **Total study population**  (N =307) | **Food secure participants**  (N = 233) | **Food insecure participants**  (N = 74) | **Total study participants**  (N =307) | **Food secure participants**  (N =233) | **Food insecure participants**  (N =74) | | |
| Age, years | 16 (5.2) | 37.5 (33.4; 42.1) | 37.0 (33.0; 41.3) | 39.0 (34.0; 43.6) | 37.4 (33.4; 42.1) | 36.9 (32.9; 41.3) | 39.0 (34.0; 43.7) | | |
| Sex, %  Male  Female | 1 (0.3) | 38 (12.4)  268 (87.6) | 27 (11.6)  205 (88.4) | 11 (14.9)  63 (85.1) | 39 (12.7)  268 (87.3) | 28 (12.0)  205 (88.0) | 11 (14.9)  63 (85.1) | | |
| Self-reported BMI^1^, kg/m^2^  Weight status^2^, %  Normal weight; BMI 18.5-24.9  Overweight; BMI 25-29.9  Obese; BMI ≥ 30 | 14 (4.6)  14 (4.6) | 27.1 (24.1; 30.5)  92 (31.4)  118 (40.3)  83 (28.3) | 26.9 (24.0; 29.8)  76 (34.1)  93 (41.7)  54 (24.2) | 28.8 (25.5; 32.7)  16 (22.9)  25 (35.7)  29 (41.4) | 27.2 (24.1; 30.5)  96 (31.3)  124 (40.4)  87 (28.3) | 26.9 (24.0; 29.8)  79 (33.9)  97 (41.6)  57 (24.5) | 28.6 (25.5; 32.6)  17 (23.0)  27 (36.5)  30 (40.5) | | |
| Gross monthly income, %  Above basic needs budget  Below basic needs budget | 19 (6.2) | 112 (38.9)  176 (61.1) | 101 (46.3)  117 (53.7) | 11 (15.7)  59 (84.3) | 125 (40.7)  182 (59.3) | 111 (47.6)  122 (52.4) | 14 (18.9)  60 (81.1) | | |
| Highest educational attainment, %  Low; ≤ ISCED 2  Medium; ISCED 3  High; ≥ ISCED 4 | 5 (1.6) | 109 (36.1)  108 (35.8)  85 (28.1) | 72 (31.6)  81 (38.5)  75 (32.9) | 37 (50.0)  27 (36.5)  10 (13.5) | 110 (35.8)  111 (36.2)  86 (28.0) | 73 (31.3)  84 (36.1)  76 (32.6) | 37 (50.0)  27 (36.5)  10 (13.5) | | |
| Current smoking, %  Yes  No | 4 (1.3) | 49 (16.2)  254 (83.8) | 30 (12.9)  202 (87.1) | 19 (26.8)  52 (73.2) | 51 (16.6)  256 (83.4) | 31 (13.3)  202 (86.7) | 20 (27.0)  54 (73.0) | | |
| Physical and mental health  PCS; range 0-100  MCS; range 0-100 | 117 (38.1)  117 (38.1) | 50.7 (42.0; 54.5)  51.5 (45.3; 57.6) | 52.2 (44.9; 54.8)  53.5 (47.0; 57.9) | 43.8 (33.4; 53.5)  46.0 (37.5; 53.7) | 44.6 (36.1; 53.5)  46.3 (36.0; 54.5) | 44.5 (36.9; 53.6)  46.2 (36.6; 55.3) | 42.5 (33.8; 49.5)  43.2 (33.8; 50.4) | | |
| Total diet quality score; range 0-100 | 0 (0.0) | 56.3 (50.1; 67.0)^3^ | 56.5 (51.0; 68.1) | 55.7 (44.5; 63.8) | 56.3 (50.1; 67.0)^3^ | 56.5 (51.0; 68.1) | 55.7 (44.5; 63.8) | | |
| Population-specific median diet quality score, %  Low diet quality; ≤56.25  High diet quality; >56.25 | 0 (0.0) | 154 (50.2)  153 (49.8) | 114 (48.9)  119 (51.1) | 40 (54.1)  34 (45.9) | 154 (50.2)  153 (49.8) | 114 (48.9)  119 (51.1) | 40 (54.1)  34 (45.9) | | |
| Currently employed, %  Yes  No | 6 (2.0) | 139 (46.2)  162 (53.8) | 117 (50.9)  113 (49.1) | 22 (31.0)  49 (59.0) | 141 (45.9)  166 (54.1) | 118 (50.6)  115 (49.4) | 23 (31.1)  51 (68.9) | | |
| Marital status, %  Single-parent household  Two-parent household | 5 (1.6) | 87 (28.8)  215 (71.2) | 59 (25.7)  171 (74.3) | 28 (38.9)  44 (59.5) | 89 (29.0)  218 (71.0) | 60 (25.8)  173 (74.2) | 29 (39.2)  45 (60.8) | | |
| Migration background, %  Western  Non-Western | 5 (1.6) | 60 (19.9)  242 (80.1) | 51 (22.3)  178 (77.7) | 9 (12.3)  64 (87.7) | 63 (20.5)  244 (79.5) | 53 (22.7)  180 (77.3) | 10 (13.5)  64 (86.5) | | |
| Religion, %  Islam  Christianity  Not religious  Other religion | 26 (8.5) | 179 (63.7)  45 (16.0)  38 (13.5)  19 (6.8) | 140 (64.8)  26 (12.0)  33 (15.3)  17 (7.9) | 39 (60.0)  19 (29.2)  5 (7.7)  2 (3.1) | 184 (60.0)  51 (16.7)  45 (14.7)  27 (8.8) | 143 (61.4)  30 (12.9)  38 (16.3)  22 (9.4) | 41 (55.4)  21 (28.4)  7 (9.4)  5 (6.8) | | |
| Household size, N | 0 (0.0) | 4.0 (3.0; 5.0) | 4.0 (3.0; 5.0) | 4.0 (3.0; 5.0) | 4.0 (3.0; 5.0) | 4.0 (3.0; 5.0) | 4.0 (3.0; 5.0) | | |
| Adult/child ratio | 0 (0.0) | 1.0 (0.5; 1.0) | 1.0 (0.5; 1.0) | 0.7 (0.5; 1.0) | 1.0 (0.5; 1.0) | 1.0 (0.5; 1.0) | 0.7 (0.5; 1.0) | | |
| Adult/child ratio, %  < 1  1  > 1 | 0 (0.0) | 153 (49.8)  106 (34.5)  48 (15.6) | 114 (48.9)  81 (34.8)  38 (16.3) | 39 (52.7)  25 (33.8)  10 (13.5) | 153 (49.8)  106 (34.5)  48 (15.6) | 114 (48.9)  81 (34.8)  38 (16.3) | 39 (52.7)  25 (33.8)  10 (13.5) | | |
| *Abbreviations: IQR, Interquartile range; N, number; BMI, Body Mass Index; ISCED, International Standard Classification of Education; PCS, Physical Component Summary; MCS, Mental Component Summary; m, meters.*  *^1^Calculated based on the self-reported body weight in kilograms (kg) divided by the self-reported body height in meters squared (m2).*  *^2^Since only two participants were classified as underweight, this category was merged with normal weight, and therefore only normal weight, overweight and obesity were reported.*  *^3^Population-specific median diet quality score.* | | | | | | | |  |  |

**Supplementary table 5** Logistic regression analysis of the associations between food insecurity status and participant characteristics within the specific layers of the socioecological model of unimputed and imputed data (N=307)

|  | **Unimputed data** | | | | **Imputed data** | | | |
| --- | --- | --- | --- | --- | --- | --- | --- | --- |
|  | **Adjusted OR** | **95% CI** | **P-value** | **Nagelkerke pseudo R^2^** | **Adjusted OR** | **95% CI** | **P-value** | **Nagelkerke pseudo R^2^** |
| ***Personal environment*** |  |  |  | *0.277* |  |  |  | *0.206* |
| **Self-reported BMI^1^, kg/m^2^** | 1.06 | (0.98; 1.15) | 0.12 |  | 1.07 | (1.00; 1.13) | 0.04*** |  |
| **Gross monthly income, %**  **Below basic needs budget**  **Above basic needs budget** | Reference  0.99 | (1.00; 1.00) | 0.31 |  | Reference  0.34 | (0.17; 0.69) | <0.01**** |  |
| **Highest educational attainment, %**  **Low; ≤ ISCED 2**  **Medium; ISCED 3**  **High; ≥ ISCED 4** | Reference  1.44  0.69 | (0.84; 2.47)  (0.40; 1.19) | 0.19  0.18 |  | Reference  0.88  0.48 | (0.46; 1.69)  (0.21; 1.15) | 0.70  0.10 |  |
| **Current smoking, %**  **Yes**  **No** | Reference  0.99 | (0.99; 1.03) | 0.52 |  | Reference  0.44 | (0.21; 0.91) | 0.03*** |  |
| **Physical and mental health**  **PCS;** range 0-100  **MCS^2^;** range 0-100  **≤ 35**  **36-46**  **47-54**  **≥ 55** | 0.96  Reference  0.833  0.469  0.299 | (0.92; 0.99)  (0.24; 2.90)  (0.15; 1.52)  (0.09; 1.01) | 0.02*  0.77  0.21  0.05 |  | 0.99  Reference  1.14  0.98  0.57 | (0.85; 1.03)  (0.39; 3.35)  (0.34; 2.78)  (0.19; 1.69) | 0.54  0.82  0.97  0.31 |  |
| **Diet quality^2^;** range: 0 – 100  **≤ 49**  **50-55**  **56-66**  **≥ 67** | Reference  0.40  0.79  0.34 | (0.12; 1.34)  (0.28; 2.28)  (0.11; 1.05) | 0.14  0.66  0.06 |  | Reference  0.53  0.89  0.53 | (0.28; 1.44)  (0.40; 1.97)  (0.23; 1.23) | 0.28  0.77  0.14 |  |
| ***Social environment*** |  |  |  | *0.139* |  |  |  | *0.140* |
| **Currently employed, %**  **Yes**  **No** | Reference  2.38 | (1.29; 4.38) | 0.01* |  | Reference  2.23 | (1.21; 4.08) | 0.01*** |  |
| **Marital status, %**  **Two-parent household**  **Single-parent household** | Reference  1.79 | (1.16; 3.32) | 0.07 |  | Reference  1.69 | (1.10; 3.13) | 0.10 |  |
| **Migration background, %**  **Western**  **Non-Western** | Reference  2.48 | (1.05; 5.85) | 0.04* |  | Reference  2.53 | (1.02; 5.90) | 0.03*** |  |
| **Religion, %**  **Islam**  **Christianity**  **Not religious**  **Other religion**  ***Physical environment***  **Liveability index^3,4^,**  **Housing stock** | Reference  3.01  0.85  0.38  0.33 | (1.57; 6.02)  (0.36; 2.04)  (0.13; 1.08)  (0.25; 0.43) | <0.01****  0.72  0.07  <0.001*** | ***0.024*** | Reference  3.12  0.86  0.37  0.33 | (1.61; 6.18)  (0.34; 2.19)  (0.13; 1.06)  (0.25; 0.43) | <0.01****  0.73  0.06  <0.001*** | ***0.024*** |
| ***Overall model*** |  |  |  | ***0.426*** |  |  |  | ***0.297*** |

*Abbreviations: OR, Odds Ratio; CI, Confidence Interval; BMI, Body Mass Index; ISCED, International Standard Classification of Education; PCS, Physical Component Summary; MCS, Mental Component Summary.*

*^1^Calculated based on the self-reported body weight in kilograms (kg) divided by the self-reported body height in meters squared (m^2^).*

*^2^Categories based on quartiles.*

*^3^Data available for N= 279.*

*^4^Index range: (1) very poor (2) poor (3) very unsatisfactory (4) unsatisfactory (5) satisfactory (6) more than satisfactory (7) good (8) very good (9) outstanding.*

**p < 0.05, **p < 0.01, ***p < 0.001 for the difference between food-insecure and food-secure households.*

**References**

1. Brink L, Postma-Smeets A, Stafleu A, van Dooren C, Wolvers D. Guidelines Wheel of Five. NNC. 2016.

2. Health Council of the Netherlands. Dutch dietary guidelines 2015. The Hague: Health Council of the Netherlands; 2015.

3. van Lee L, Feskens EJ, Meijboom S, van Huysduynen EJH, van’t Veer P, de Vries JH, et al. Evaluation of a screener to assess diet quality in the Netherlands. Br J Nutr. 2016;115(3):517-26.

4. van der Velde LA, Nyns CJ, Engel MD, Neter JE, van der Meer IM, Numans ME, et al. Exploring food insecurity and obesity in Dutch disadvantaged neighborhoods: a cross-sectional mediation analysis. BMC Public Health. 2020;20(1):569.

5. Parmenter K, Waller J, Wardle J. Demographic variation in nutrition knowledge in England. Health education research. 2000;15(2):163-74.

6. Hansbro J, Bridgwood A, Morgan A, Hickman M. Health in England 1996: what people know, what people think, what people do: Stationery Office London, England; 1997.

7. Statistics Netherlands (CBS). Trends in the Netherlands 2017. [Internet]. The Hague: CBS; 2017 [cited 2021 Jan 02]. Available from: <https://longreads.cbs.nl/trends17/arbeid_en_inkomen/trends/>.

8. Coltrane S. Research on household labor: Modeling and measuring the social embeddedness of routine family work. Journal of Marriage and family. 2000;62(4):1208-33.

9. DeVault ML. Feeding the family: The social organization of caring as gendered work: University of Chicago Press; 1994.

10. Hendrie GA, Coveney J, Cox D. Exploring nutrition knowledge and the demographic variation in knowledge levels in an Australian community sample. Public Health Nutrition. 2008;11(12):1365-71.

11. Wardle J, Parmenter K, Waller J. Nutrition knowledge and food intake. Appetite. 2000;34(3):269-75.

12. Kim TJ, von dem Knesebeck O. Income and obesity: what is the direction of the relationship? A systematic review and meta-analysis. BMJ open. 2018;8(1):e019862.

13. Campos-Vazquez RM, Gonzalez E. Obesity and hiring discrimination. Economics & Human Biology. 2020;37:100850.

14. Pelzer B, Schaffrath S, Vernaleken I. Coping with unemployment: The impact of unemployment on mental health, personality, and social interaction skills. Work. 2014;48(2):289-95.

15. Kim SS, Muntaner C, Kim H, Jeon CY, Perry MJ. Gain of employment and depressive symptoms among previously unemployed workers: a longitudinal cohort study in South Korea. American journal of industrial medicine. 2013;56(10):1245-50.

16. Oliffe JL, Han CS. Beyond workers’ compensation: Men’s mental health in and out of work. American journal of men's health. 2014;8(1):45-53.

17. Demerouti E, Bakker AB, Nachreiner F, Schaufeli WB. The job demands-resources model of burnout. Journal of Applied psychology. 2001;86(3):499.

18. Drammeh W, Hamid NA, Rohana A. Determinants of household food insecurity and its association with child malnutrition in Sub-Saharan Africa: a review of the literature. Current Research in Nutrition and Food Science Journal. 2019;7(3):610-23.

19. Dallongeville J, Marécaux N, Cottel D, Bingham A, Amouyel P. Association between nutrition knowledge and nutritional intake in middle-aged men from Northern France. Public Health Nutrition. 2001;4(1):27-33.

20. Antonides G, De Groot I, van Raaij WF. Resultaten financieel inzicht van Nederlanders: Publieksonderzoek over 4280 consumenten uitgevoerd in opdracht van CentiQ. CentiQ; 2008.

21. Worsley A. Nutrition knowledge and food consumption: can nutrition knowledge change food behaviour? Asia Pacific journal of clinical nutrition. 2002;11:S579-S85.

22. Marcus AC, Shopland DR, Crane LA, Lynn WR. Prevalence of cigarette smoking in the United States: estimates from the 1985 current population survey. JNCI: Journal of the National Cancer Institute. 1989;81(6):409-14.

23. Barnett R, Pearce J, Moon G. Community inequality and smoking cessation in New Zealand, 1981–2006. Social science & medicine. 2009;68(5):876-84.

24. Rasmussen SR, Prescott E, Sørensen TI, Søgaard J. The total lifetime costs of smoking. The European Journal of Public Health. 2004;14(1):95-100.

25. Fulkerson JA, Larson N, Horning M, Neumark-Sztainer D. A review of associations between family or shared meal frequency and dietary and weight status outcomes across the lifespan. Journal of Nutrition Education and behavior. 2014;46(1):2-19.

26. Perreira KM, Crosnoe R, Fortuny K, Pedroza J, Ulvestad K, Weiland C, et al. Barriers to immigrants’ access to health and human services programs. ASPE Issue Brief Washington, DC: Office of the Assistant Secretary for Planning and Evaluation. 2012.

27. Statistics Netherlands (CBS). Income: CBS; 2016 [cited 2021 Feb 05]. Available from: <https://www.cbs.nl/nl-nl/achtergrond/2016/47/inkomen>.

28. Karimshah A, Chiment M, Skrbis Z. The mosque and social networks: The case of Muslim youth in Brisbane. Social Inclusion. 2014;2(2):38-46.

29. Gallet W. Social Connectedness: The role of the local church in building community. Pointers: Bulletin of the Christian Research Association. 2016.

30. Short M. Three Anglican churches engaging with people from culturally and linguistically diverse backgrounds: Bush Church Aid Society; 2015.

31. Van Der Velde LA, Schuilenburg LA, Thrivikraman JK, Numans ME, Kiefte-de Jong JC. Needs and perceptions regarding healthy eating among people at risk of food insecurity: a qualitative analysis. International journal for equity in health. 2019;18(1):184.

32. Statistics Netherlands (CBS). Healthier food will increase in price more than unhealthy food [Internet]. CBS; 2018 [cited 2021 Jan 06]. Available from: <https://www.cbs.nl/nl-nl/nieuws/2018/03/gezonder-eten-stijgt-meer-in-prijs-dan-ongezonder-eten>.

33. Ali Naser I, Jalil R, Wan Muda WM, Wan Nik WS, Mohd Shariff Z, Abdullah MR. Association between household food insecurity and nutritional outcomes among children in Northeastern of Peninsular Malaysia. Nutrition research and practice. 2014;8(3):304-11.

34. Walen HR, Lachman ME. Social support and strain from partner, family, and friends: Costs and benefits for men and women in adulthood. Journal of social and personal relationships. 2000;17(1):5-30.

35. Jacobs JA, Gerson K. Overworked Individuals or Overworked Families?:Explaining Trends in Work, Leisure, and Family Time. Work Occup. 2001;28(1):40-63.

36. Statistics Netherlands (CBS). Workers. [Internet]. CBS; [cited 2021 Feb 03]. Available from: <https://www.cbs.nl/nl-nl/visualisaties/dashboard-arbeidsmarkt/werkenden>.

37. Ihab A, Rohana A, Manan WW, Suriati WW, Zalilah M, Rusli AM. Nutritional outcomes related to household food insecurity among mothers in rural Malaysia. Journal of Health, Population, and Nutrition. 2013;31(4):480.

38. Bogale A, Shimelis A. Household level determinants of food insecurity in rural areas of Dire Dawa, Eastern Ethiopia. African Journal of Food, Agriculture, Nutrition and Development. 2009;9(9).

39. Hanson KL, Connor LM. Food insecurity and dietary quality in US adults and children: a systematic review. The American journal of clinical nutrition. 2014;100(2):684-92.

40. Schneider S, Gruber J. Neighbourhood deprivation and outlet density for tobacco, alcohol and fast food: first hints of obesogenic and addictive environments in Germany. Public health nutrition. 2013;16(7):1168-77.

41. Pearce J, Day P, Witten K. Neighbourhood provision of food and alcohol retailing and social deprivation in urban New Zealand. Urban Policy and Research. 2008;26(2):213-27.

42. Ball K, Salmon J, Giles-Corti B, Crawford D. How can socio-economic differences in physical activity among women be explained? A qualitative study. Women & health. 2006;43(1):93-113.

43. Ellaway A, Macintyre S, Bonnefoy X. Graffiti, greenery, and obesity in adults: secondary analysis of European cross sectional survey. Bmj. 2005;331(7517):611-2.

44. Rejineveld S. The impact of individual and area characteristics on urban socioeconomic differences in health and smoking. International journal of epidemiology. 1998;27(1):33-40.
